# Control Strategies for Endemic Childhood Scabies

**DOI:** 10.1371/journal.pone.0015990

**Published:** 2011-01-25

**Authors:** Stephen J. Gilmore

**Affiliations:** Dermatology Research Centre, School of Medicine, Princess Alexandra Hospital, The University of Queensland, Brisbane, Australia; Kenya Medical Research Institute - Wellcome Trust Research Programme, Kenya

## Abstract

Human scabies is a major global public health issue, with an estimated 300 million cases per year worldwide. Prevalence rates are particularly high in many third-world regions and within various indigenous communities in developed countries. Infestation with *Sarcoptes Scabiei* is associated with group-A streptococcal pyoderma which in turn predisposes to rheumatic fever, acute glomerulonephritis and their respective long-term sequelae: rheumatic heart disease and chronic renal insufficiency. The documented difficulties inherent in achieving scabies control within affected communities have motivated us to develop a network-dependent Monte-Carlo model of the scabies contagion, with the dual aims of gaining insight into its dynamics, and in determining the effects of various treatment strategies. Here we show that scabies burden is adversely affected by increases in average network degree, prominent network clustering, and by a person-to-person transmissibility of greater magnitude. We demonstrate that creating a community-specific model allows for the determination of an effective treatment protocol that can satisfy any pre-defined target prevalence. We find frequent low-density treatment protocols are inherently advantageous in comparison with infrequent mass screening and treatment regimes: prevalence rates are lower when compared with protocols that administer the same number of treatments over a given time interval less frequently, and frequent low-density treatment protocols have economic, practical and public acceptance advantages that may facilitate their long-term implementation. This work demonstrates the importance of stochasticity, community structure and the heterogeneity of individuals in influencing the dynamics of the human scabies contagion, and provides a practical method for investigating the outcomes of various intervention strategies.

## Introduction

Human scabies, due to infestation with the mite *Sarcoptes Scabiei var Hominis*, affects an estimated 300 million people worldwide per year and has a long documented history of affecting human populations [1]. While increased prevalence rates are usually associated with poor sanitation and overcrowding [2], disease burden may also increase in times of mass human migration, as occurred in Europe during both world wars [3]. Overcrowding without poor sanitation – a previously reported community characteristic of the Kuna Indians residing in small islands off the coast of Panama – may also lead to high scabies burdens [4]. Scabies may also occur as epidemics in hospitals, nursing homes and long-term care facilities [1].

Although endemic in most extant human populations, prevalence and incidence rates are particularly high in many third-world regions and within various indigenous communities in developed countries [2], [4], [5], [6], [7], [8]. Within these demographic groups the high prevalence rates of childhood scabies – which have been variously reported as 100% [1], 18.5% [5], 25% [6] and 50% [8] – are strongly associated with increased prevalence rates of group-A streptococcal impetigo [8]. Children affected by recurrent cutaneous pyoderma are at significant risk of developing rheumatic fever or acute glomerulonephritis and their possible sequelae: rheumatic heart disease or chronic renal insufficiency [8], [9], [10]. Heightened awareness of the morbidity associated with chronic cardiac and renal disease has prompted health care workers worldwide to develop, and in some cases, implement, local community health programs aimed at reducing the long-term scabies burden [6], [7], [11], [12].

Currently there exists limited evidence for the effective, long-term management of scabies in communities with hyper-endemic prevalence rates [13]. While mass treatment of affected communities has been advocated as the only sensible method of achieving control, other possible strategies, such as the random treatment of affected individuals, have been dismissed [14].

Although community-wide treatment with topical 1% gamma benzene hexachloride [4], topical 5% permethrin [14] or oral ivermectin [6] have produced long-term reductions in scabies burdens, these efforts require ongoing mass screening of entire populations and treatment of new cases to keep prevalence rates low. When screening is temporarily halted, prevalence rates quickly escalate [14]. Given that these interventions require high levels of community participation and enthusiasm (which may diminish as scabies burden decreases), it is unclear whether such protocols can be maintained indefinitely. Furthermore, external factors may preclude the delivery of long-term control. While fixed-term funding and other financial constraints are commonplace, adverse unforeseen events can also occur: for example, the American invasion of Panama in 1989 stopped the screening program implemented among the Kuna Indians in the San Blas archipelago off the coast of Central America [14]. Less intensive programs, such as the once-off widespread topical treatment of index cases and the unsupervised treatment of their immediate household contacts have yielded unsatisfactory outcomes, probably because of poor compliance [12], [15]. Low density continuous treatment strategies have also failed: among a population of 2000 individuals living in a San Blas island off the coast of Panama, the treatment of 30 cases per week for two years did not reduce scabies prevalence below 50% [4].

Taken together, the considerations above highlight the difficulties in achieving scabies control in communities where overcrowding and poverty continue to exist. While many authors have called for further epidemiologic research, others have highlighted the pressing need for more basic and practical advances. Such advances may include, for example, improved understanding of the immunologic factors associated with infestation, the development of clinical immuno-diagnostics, or the realisation of safe and effective therapies for infants [2], [8]. For a discussion of the relative merits of the two major treatment modalities – topical permethrin and oral ivermectin, and the threat of resistance to either of these agents – we refer the reader to the references herein [6], [16], [17], [18], [19]. We also note the problems associated with the diagnosis of scabies; both the fluid nature of the health care workforce and the variable clinical skill level available in remote and poor communities will at times lead to both under and over diagnosis.

In the following sections we take an alternative yet complimentary approach to the problem of scabies epidemiology and present an agent-based model of disease burden in a simulated community. This work is driven in part by its context within the broader discipline of infectious disease modelling; indeed, mathematical models can provide a framework that enhance understanding of the complex dynamics of infectious disease epidemics [20], which in turn may facilitate the design of effective intervention and control strategies [21]. Our motivation to simulate the human scabies contagion is therefore twofold: first, to use our model to gain insights into its community-wide dynamics, and second, to develop a methodology that can facilitate the determination of practical and effective control strategies in communities with high scabies loads, given the conflicts associated with desirable outcome measures on one hand, and the constraints of cost, limited public health infrastructure and compliance issues on the other. The remainder of this paper describes how we have achieved these goals; in particular, our results demonstrate the importance of stochasticity, community structure and the heterogeneity of individuals in influencing the dynamics of the human scabies contagion, and we provide a method for calculating practical and effective treatment regimes. We believe public health authorities and aid workers involved in the development of scabies control policy and in the design and implementation of treatment protocols may benefit by utilising insights gained from our approach.

## Methods

Here we present an agent-based model of the human scabies contagion. All simulations were performed using code written for *Mathematica 7.0* running on a Macintosh G4 with 4MB of RAM.

### Community structure

**Figure 1:**
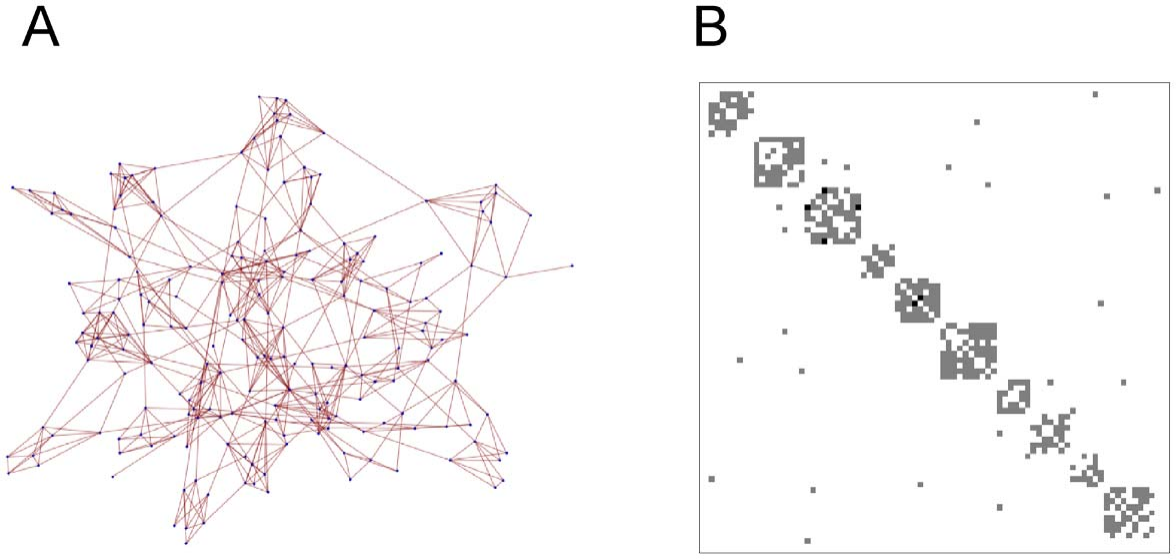
Network structure and connectivity matrix. In (a), a typical example of the 200-vertex network architecture used to model community structure is shown. In (b) we represent the connectivity between the first 80 vertices of this network as a connectivity matrix, where grey squares represent connections between pairs of vertices. Note the clustering (represented as higher connectivity along the main diagonal), the symmetry (the non-directed nature of the interaction between vertices) and its overall sparseness (most pairs of vertices remain unconnected).

**Figure 2:**
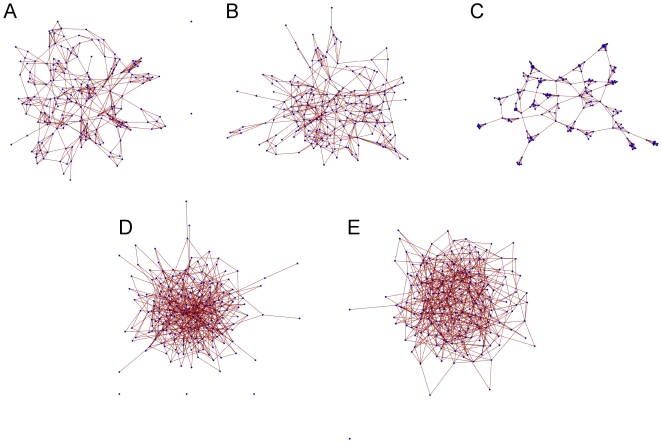
Examples of small-world network architectures. In (a), we represent the expected community structure with a non-directed graph of 200 vertices that is clustered and exhibits a small number of vertices with multiple connections. The average degree is ∼6, indicating individuals are in close contact with an average of 6 people. In (b), we decrease the average degree to ∼4 while maintaining the architectural features of (a). In (c), the highly connected vertices are absent hence all individuals are regularly connected. In (d) and (e) all local clustering is lost: (d) is representative – given the constraint of a relatively small network size – of a scale-free structure, while (e) is a regular random graph. Note that we have constructed (c), (d) and (e) such that the average degree is the same as (a). Each class of graph is generated randomly and determined by the values of three adjustable parameters (see text).

We abstract community structure as a static non-directed graph where the vertices represent individuals and the edges the close physical association of one individual with another. We define the physical association between two people as close if they are either sharing a household or classroom. We generate our graphs by constructing sparse symmetric connectivity matrices (Fig. 1) with properties defined by three adjustable parameters: first, the probability of a non-zero entry at any matrix position (corresponding to the connectivity^1^); second, the probability of spread of non-zero entries centred about the main diagonal (corresponding to the degree of clustering^2^); and third, a *Zipf* probability that determines the distribution of non-zero entries of each row of the matrix (corresponding to the degree distribution^3^) (Fig. 2). Each vertex is a binary variable that identifies that person as either carrying or not carrying the scabies mite. Since there is an over-representation of children and young adults less than 20 years of age in many indigenous communities worldwide, including indigenous Australians [22], we define the age distribution of the population as a truncated Normal distribution, peaking at about age 20 [22], and with a maximum around 50.

### Updating scheme

**Figure 3:**
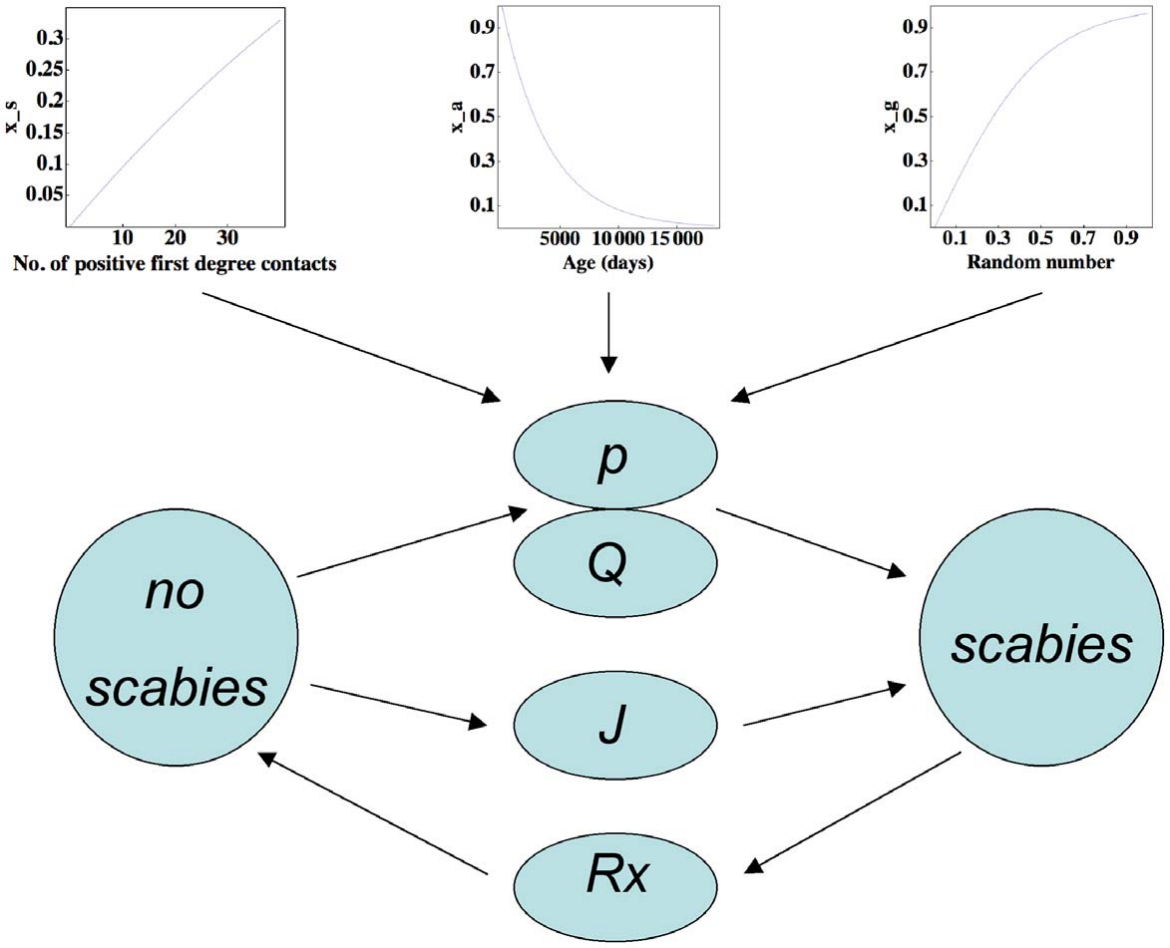
The updating rules. The decision algorithm determining whether a non-affected individual within the community acquires scabies or whether an affected person is treated is shown in schematic form. The probability of developing scabies depends on the number of first-degree contacts that are positive (governed by the relation shown at top left), the individuals' age (top centre relation) and their genetic susceptibility (top right relation), in addition to *Q*, the community-wide acquisition probability, and *J*, the probability of acquiring scabies from person(s) external to the community. Treatment probabilities (*Rx*) are defined in terms of density and frequency.

We model the evolution of the population using a Monte-Carlo updating scheme, and for the simulation results reported here, we track its evolution daily over a twelve-month period. At each iteration, a vertex is chosen at random for updating (Fig. 3). If that vertex represents an individual without scabies, then the probability of developing scabies depends, in a stochastic manner, on five main variables: a community-wide transmissibility parameter *Q* that quantifies the likelihood of scabies acquisition, the number of first-degree links that are positive for scabies, the individual's age and genetic susceptibility, and the importation likelihood *J*. Mathematically, the probability of developing scabies, at each Monte-Carlo round, is given by *Q p* + *J <1* where 
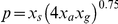
. Here 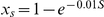 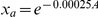 and 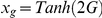 where *S* is the number of first-degree contacts positive for scabies, *A* is the age of the individual in days, and *G* is a number randomly drawn from the uniform interval *(0, 1)* giving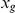 as a measure of genetic susceptibility. While *x_a_* is a monotonically decreasing function (corresponding to the diminishing probability of scabies acquisition with age), both *x_s_* and *x_g_* are monotonically increasing. While *x_s_* flattens as the number of first-degree positive contacts becomes very large, the genetic susceptibility of the population *x_g_* is skewed: fewer people are highly resistant to scabies acquisition than are highly sensitive (See Fig. 3).

The individual-dependent factor *p* is designed to assume values that range from zero (corresponding to no first degree contacts affected by scabies) to one (corresponding to a realistic maximum of 35 first degree contacts positive for scabies, an age less than 1 day, and the genetic predisposition to scabies acquisition at or above the 99^th^ centile). For simplicity, we do not vary any numerical parameters within *p* in any of the simulations reported here. These parameters serve to fix the relative importance of each variable and determine the qualitative relation between input and output. Of course, it is possible to develop alternative functions or parameter values for the elements within *p*; however, we have chosen our particular functions to be simple yet plausible. For example, consider the following two cases: first, when the number of first-degree contacts is less than 10, *x_s_* is nearly linear, indicating that doubling the number of positive first-degree contacts doubles the product *Q p* – and thus the probability of within-community scabies acquisition; and second, if the age of the individual increases from 10 to 20 years, the product *Q p* is approximately halved, reducing the probability of within-community scabies acquisition by 50% (See Fig. 3).

If the randomly chosen vertex represents an individual with scabies, we define treatment as the only way in which that individual can be cured, which we call an *effective treatment*. While we specify treatment densities and frequencies exactly, affected individuals (with or without their first degree contacts) are chosen at random.

### Implementation

To implement our model, and to simplify our search through parameter space, we begin by developing alternative models that correspond, respectively, to a parameter regime with model output that reproduces the documented *prevalence* and *incidence* rate of a community with a high scabies burden [5], and a parameter regime with model output that reproduces the documented *prevalence* and *treatment* rate of a community with hyper-endemic scabies [4]. For the first case where the treatment rate is not reported we specify the treatment density and frequency of index cases only; the total effective treatment rate is not *a priori* determined exactly since all first degree contacts, irrespective of whether they have scabies, are also treated. For the second case, where field data provides accurate treatment rates, we specify the treatment density and frequency of randomly selected individuals with scabies only, without treating any of their first-degree contacts. Taking these two cases (which we label as scenarios 1 and 2) as independent baseline models, we vary our parameters one at a time to investigate their relative importance with respect to scabies burden. Note that at this stage we are not comparing the two treatment regimes directly – the two scenarios are at present not strictly comparable since the magnitude of *Q* for scenario 1 is five times less that its corresponding value in scenario 2.

We first investigate the possible effects of different network sizes and architectures on our models' behaviour. Our baseline networks comprise 200 vertices and are small-world, clustered and exhibit a degree distribution with a longer tail in comparison with a regular degree distribution: examples are shown in Figs. 1a and 2a. These architectures are likely to most accurately capture the connections between individuals in human societies [23], [24]. To investigate the possible influence of network size on scabies dynamics, we ran multiple simulations with this architecture, for both scenarios described above, at network sizes 100, 500 and 1000. In all cases, the mean prevalence and incidence rates of scabies within those simulated communities was found to be the same as our 200-vertex network, giving us confidence that our model is robust to changes in network size, and permitting us to focus, for the remainder of our simulations, on networks of size 200. Limiting the size of our networks allows us to complete all simulations in reasonable computer time.

We consider four other types of small-world network structure: clustered with a degree distribution similar to baseline but with a lower average degree (Fig. 2b), clustered with regular links between the clusters (Fig. 2c), approximately scale-free without clustering (Fig. 2d), and regular without clustering (Fig. 2e). We next investigate the effects of varying *Q* and *J*. These community-wide parameters will depend, among other factors, on inter-community socialisation levels, societal behavioural characteristics, clothing practices, the presence or absence of impetigo, and the nature of the domestic and school environment.

To investigate the effects of different treatment densities and frequencies on the prevalence and incidence of scabies, we first use our Monte-Carlo approach and then develop a mean-field approximation. (Box 1). We are motivated to develop a mean-field approach on two counts: first, if we can demonstrate close qualitative and quantitative behaviour between the mean-field and stochastic models, then it may be far simpler to implement predictions based on analytic calculations rather than running full simulations. Second, if there is disagreement between the two models, either qualitative or quantitative, then the implications will be twofold: first, such a result will highlight the difficulties in obtaining an accurate analytic representation of the scabies contagion; and second, it will emphasise the importance of stochasticity, community structure and individual heterogeneity in scabies epidemiology.

To conclude, we compare our alternative treatment strategies directly. As noted above, these strategies correspond to either treating index cases and all first-degree contacts, or treating index cases only.

## Results

### General features

**Figure 4:**
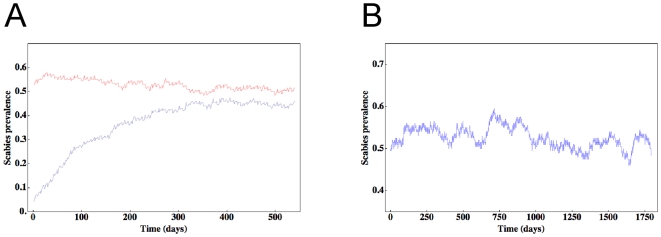
Scabies prevalence. Here we show daily prevalence rates for scenario 2 over a 36-month period (a). Beginning with arbitrary scabies prevalence rates of 0.04 and 0.54, note all relaxation times are ∼300 days. Both simulations thus converge and remain at steady-state with stochastic fluctuations. In (b), we track the evolution of a single run of scenario 2 daily and at equilibrium over 5 years, with a magnified prevalence scale to highlight the fluctuations. However, despite the fluctuations, note the time series is stationary; for example, the mean and variance of these data for years 2 and 5 are 0.53 and 0.51, and 4.4×10^−4^ and 3.6×10^−4^ respectively.

We first note the *diffusion times* – corresponding to the transient times to equilibrium for the spread or diminution of scabies throughout our networks – were generally of the order 12 months hence in the analyses that follow scabies prevalence and incidence rates were calculated after 12 months of lead-in time. This feature of the model is illustrated in Fig. 4a for scenario 2: beginning with arbitrary initial prevalence rates we found both simulations reached their steady states approximately 12 months later. We also note that our scabies model can be considered a stationary process since the mean and variance do not change over time. This feature is illustrated in Fig. 4b: here we track the evolution of scabies prevalence for scenario 2 at equilibrium over 5 years. Results from Tables 1, 2 and 3 are given as the means and 3 standard deviations of 24 independent year-long simulations. Although the fluctuations in mean prevalence rates are larger for smaller network sizes, the means are equivalent for *any* network size. Given equivalent means, and coupled to the observation that the standard deviation drops to less than 5% of the mean for network sizes of at least 500, we are confident that 24 independent year-long simulations will yield adequate statistics.

**Table 1:** Scenario 1 – treatment regime based on treating one index case and all first degree contacts every seven days (*Q* = 10^−1.5^).

		Network size			Network structure			
	BL	100	500	1000	A	B	C	D
**Network statistics**								
*Degree*	**6.0, 1.3**	6.1, 1.4	6.1, 0.5	5.8, 0.3	4.6, 1.0	6.2, 0.7	6.1, 1.9	5.9, 0.7
*Max. degree*	**21.6, 29.0**	22.8, 38.4	20.3, 36.9	21.4, 23.0	25.9, 48.0	12, 7.9	48, 79.8	13.0, 2.9
*Connectivity*	**0.03, 0.01**	0.06, 0.01	0.01, 0.00	0.01, 0.00	0.02, 0.01	0.03, 0.00	0.03, 0.01	0.03, 0.00
*Clustering Coeff.*	**0.37, 0.13**	0.30, 0.12	0.50, 0.12	0.53, 0.06	0.20, 0.11	0.68, 0.10	0.04, 0.03	0.03, 0.02
*Expected CC*	**0.03, 0.01**	0.06, 0.01	0.01, 0.00	0.01, 0.00	0.02, 0.01	0.03, 0.01	0.03, 0.01	0.03, 0.01
**Scabies results**								
*All prevalence*	**0.15, 0.08**	0.14, 0.11	0.16, 0.03	0.18, 0.03	0.09^*^, 0.09	0.18, 0.08	0.09^*^, 0.06	0.11^*^, 0.06
*<10 yo prevalence*	**0.26, 0.19**	0.27, 0.24	0.27, 0.09	0.30, 0.07	0.16^*^, 0.16	0.29, 0.17	0.16^*^, 0.13	0.20^*^, 0.07
*All incidence* **	**36.2, 6.6**	37.4, 17.3	35.1, 6.0	33.4, 0.4	30.2, 8.6	34.5, 7.2	36.1, 6.7	38.3, 6.0
*<10 yo incidence*	**60.2, 19.7**	68.2, 58.1	58.7, 21.7	58.1, 1.5	54.7, 19.2	58.5, 22.6	62.5, 18.6	68.3, 19.2
*All effective Rx* ***	**35.3, 5.7**	37.5, 15.4	35.0, 3.5	33.9, 0.3	31.9, 5.5	34.4, 4.3	37.4, 5.2	38.1, 5.5
*<10 yo effective Rx*	**58.6, 21.9**	68.3, 59.8	59.5, 19.5	57.8, 1.2	56.3, 16.4	60.3, 21.2	66.1, 16.9	67.5, 29.1

Results of simulating our model at equilibrium as a function of varying network size and structure. All results are given as the means and three standard deviations of 24 individual one-year simulations at equilibrium. Each simulation is based on a randomly generated network of the given architecture. For prevalence rates only, results denoted by an asterix indicate mean values significantly different from the mean values given in the baseline column. (Student's *t*-test with significance accepted if *p*<0.001).

BL: Baseline network (Boldface, *N* = 200, clustered, broad degree distribution).

A: *N* = 200; clustered, broad degree distribution, low degree.

B: *N* = 200; clustered, regular.

C: *N* = 200; Non-clustered, approximately scale free.

D: *N* = 200; Non-clustered, regular.

*Expected CC*: The clustering coefficient of a randomly generated graph with the same connectivity.

**Incidence rates per 100 person years.

***Effective treatments per 100 person years.

**Table 2:** Scenario 2 – treatment regime based on treating three index cases, without treating any first-degree contacts, every seven days (*Q* = 5×10^−1.5^).

		Network size			Network structure			
	BL	100	500	1000	A	B	C	D
**Network statistics**								
*Degree*	**6.0, 1.1**	5.9, 1.6	6.0, 0.5	5.9, 0.3	4.6, 1.2	6.2, 0.7	6.1, 2.2	5.8, 0.6
*Max. degree*	**19.3, 21.2**	18.8, 15.4	21.4, 40.1	20.5, 16.4	22.8, 58.6	12.8, 17.2	42.4, 80.4	13.0, 3.7
*Connectivity*	**0.03, 0.01**	0.06, 0.02	0.01, 0.00	0.01, 0.00	0.02, 0.01	0.03, 0.00	0.03, 0.01	0.03, 0.00
*Clustering Coeff.*	**0.36, 0.12**	0.30, 0.13	0.50, 0.12	0.53, 0.04	0.20, 0.11	0.68, 0.13	0.04, 0.03	0.03, 0.01
*Expected CC*	**0.03, 0.01**	0.05, 0.01	0.01, 0.00	0.01, 0.00	0.02, 0.01	0.03, 0.01	0.03, 0.01	0.03, 0.01
**Scabies results**								
*All prevalence*	**0.48, 0.12**	0.48, 0.17	0.50, 0.06	0.51, 0.04	0.37^*^, 0.14	0.50, 0.10	0.45, 0.14	0.46, 0.10
*<10 yo prevalence*	**0.67, 0.14**	0.66, 0.24	0.68, 0.07	0.69, 0.06	0.56^*^, 0.17	0.70, 0.16	0.61^*^, 0.16	0.64, 0.13
*All incidence* **	**77.9, 4.5**	76.0, 19.4	79.2, 8.6	75.2, 6.8	77.5, 6.8	76.8, 6.2	77.8, 5.3	77.6, 5.9
*<10 yo incidence*	**109, 16.4**	104, 46.9	109, 27.2	103, 13.8	116, 22.3	100, 22.6	106, 13.8	109, 18.6
*Total effective Rx* ***	**75.7, 1.1**	77.4, 2.1	77.4, 1.1	75.9, 0.5	75.9, 1.0	75.6, 0.9	76.0, 1.0	76.0, 0.7
*<10 yo effective Rx*	**108, 15.5**	110, 50.4	108, 24.0	104, 12.9	114, 20.1	105, 22.6	103, 20.0	108, 24.1

Results of simulating our model at equilibrium as a function of varying network size and structure. All results are given as the means and three standard deviations of 24 individual one-year simulations at equilibrium. Each simulation is based on a randomly generated network of the given architecture. For prevalence rates only, results denoted by an asterix indicate mean values significantly different from the mean values given in the baseline column. (Student's *t*-test with significance accepted if *p*<0.001).

BL: Baseline network (Boldface, *N* = 200, clustered, broad degree distribution).

A: *N* = 200; clustered, broad degree distribution, low degree.

B: *N* = 200; clustered, regular.

C: *N* = 200; Non-clustered, approximately scale free.

D: *N* = 200; Non-clustered, regular.

*Expected CC*: The clustering coefficient of a randomly generated graph with the same connectivity.

**Incidence rates per 100 person years.

***Effective treatments per 100 person years.

**Table 3:** Effects of different treatment protocols on scabies burden.

Scenario 1*Q* = 10^−1.5^	Max. Rx density *(index cases/200 persons), and all first degree contacts*			
	1	4	12	36
**Rx frequency** ***(days^−1^)***				
**5**	**0.08**, 38.6, 41.4, 4900.14, 69.0, 73.4	**0.04**, 45.5, 45.6, 6410.01, 85.0, 85.3	**0.04**, 44.6, 44.9, 5910.01, 87.1, 87.3	**0.04**, 45.8, 46.1, 6450.01, 88.6, 88.8
**20**	**0.29**, 24.6, 21.5, 1280.47, 36.9, 34.5	**0.09**, 40.9, 42.7, 4380.15, 72.8, 77.3	**0.01**, 43.1, 43.0, 5200.03, 88.0, 87.4	**0.01**, 44.5, 45.3, 5530.02, 80.1, 81.4
**60**	**0.41**, 20.7, 10.0, 450.57, 27.2, 15.7	**0.28**, 28.6, 24.2, 1480.44, 45.2, 39.7	**0.09**, 39.0, 41.2, 4440.17, 69.2, 73.0	**0.04**, 43.4, 44.7, 5230.07, 78.6, 79.2
**180**	**0.48**, 18.6, 3.5, 140.67, 21.0, 4.9	**0.40**, 22.0, 12.1, 490.57, 31.1, 16.7	**0.28**, 28.0, 24.0, 1490.44, 42.8, 39.6	**0.13**, 37.7, 40.3, 4510.24, 68.1, 69.5

Scenario 2*Q* = 5×10^−1.5^	Max. Rx density *(index cases/200 persons)*			
	3	10	30	90
**Rx frequency** ***(days^−1^)***				
**6**	**0.43**, 89.4, 89.1,1790.61, 127, 126	**0.02**, 208, 209, 4180.04, 403, 405	**0.02**, 209, 210, 4200.04, 412, 415	**0.02**, 201, 202, 4040.04, 401, 403
**20**	**0.70**, 34.5, 26.8, 540.86, 34.9, 32.0	**0.43**, 89.0, 89.2, 1790.62, 128, 127	**0.06**, 191, 192, 3840.11, 366, 367	**0.06**, 194, 195, 3900.11, 367, 367
**60**	**0.79**, 18.6, 8.9, 180.93, 12.5, 10.0	**0.69**, 35.6, 29.8, 600.84, 38.1, 36.3	**0.45**, 89.8, 89.2,1790.64, 127, 126	**0.15**, 162, 164, 3280.28, 278, 280
**180**	**0.83**, 13.5, 3.0, 60.95, 7.8, 3.5	**0.80**, 19.1, 9.9, 200.92, 15.3, 12.1	**0.70**, 36.2, 29.9, 600.86, 40.0, 37.1	**0.44**, 88.2, 89.2, 1790.63, 121, 122

Shown in the first row of each box are results for all age groups, while the second row of each box shows results for the 0–10 year age group. The first row within each box shows the prevalence (boldface) and incidence rates of scabies (per 100 person-years), in addition to the total effective treatment rate (per 100 person years) and the total treatment count over 360 days. The second row within each box shows the prevalence and incidence rates in the 0–10 age group (per 100 person-years)**,** and the treatment rate (per 100 person-years)**.** Entries for each box are averages generated from the results of 24 one-year simulations.

### Scenarios 1 and 2

**Figure 5:**
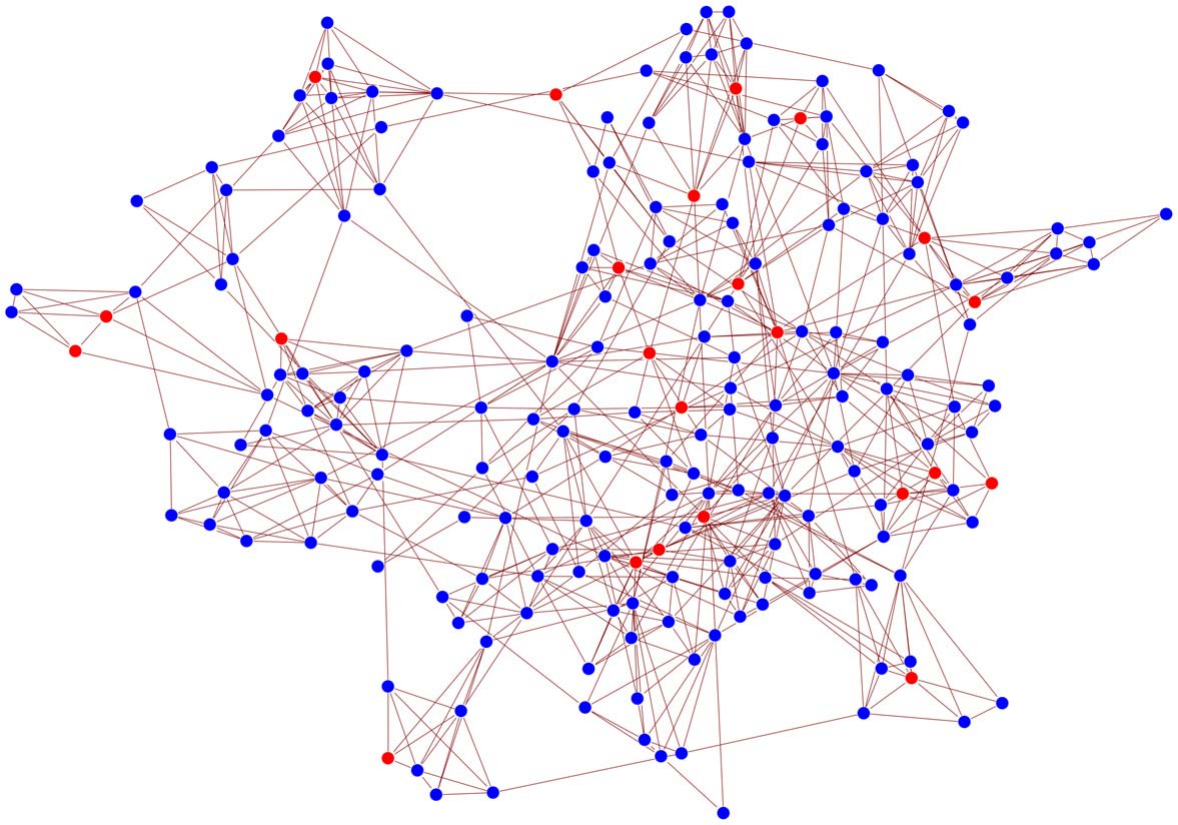
Scabies clustering. Typical example from scenario 1 of a 200-node network at equilibrium showing affected individuals (red disks) in the context of community structure. Note how scabies occurs in clusters.

Results from our baseline networks are shown in the first column of Tables 1 and 2. We note in Table 1 that the average prevalence and incidence rate for scabies among the 0 to 10 age group are 26% and 60 per 100 child-years respectively: results that are in good agreement with reported field data from Fiji [5]. For this case, Fig. 5 shows an example of the typical clustered distribution of scabies cases within the context of community structure. The first column of Table 2 reveals an overall prevalence of scabies of 48% despite a total of 154 effective treatments per year. This scenario corresponds to the documented report among 2070 individuals, where, despite 30 treatments per week for two years, scabies prevalence remained near 50% [4]. Both scenarios revealed the mean and median age of the population affected by scabies are significantly less than their respective values for the whole population.

While the mean prevalence and incidence rates of scabies in our model were independent of network size (when other network variables are controlled; see Tables 1 and 2), we found that two architectural features significantly influence scabies burden: the networks' average degree and the presence or absence of clustering. In these cases, a larger average degree or an elevated clustering coefficient are independently associated with scabies burdens of greater magnitude. We found that the degree distribution of community structure does not influence scabies burden (Tables 1 and 2).

**Figure 6:**
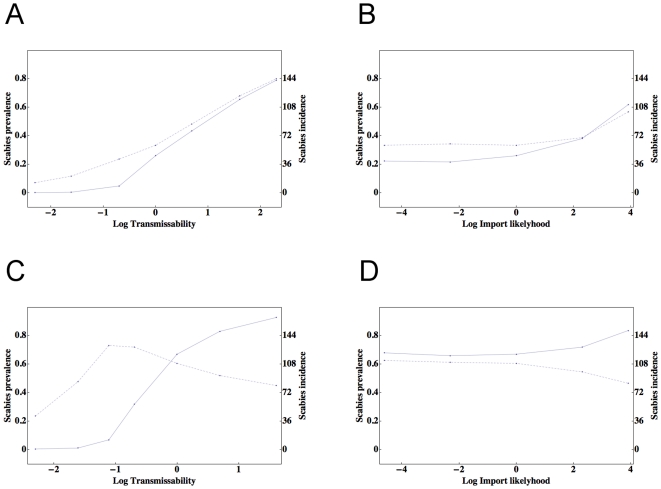
Prevalence and incidence rates for scenario 1 (a, b) and 2 (c, d). Effects on prevalence and incidence rates per 100 child-years (solid and dashed lines, respectively) of scabies in the 0–10 year-old age group as a function of the natural logarithm of the transmissibility *Q* (a, c), and as a function of the natural logarithm of the likelihood of importation *J* (b, d). For convenience, baseline transmissibility and the probability of importation are rescaled to the value 1 hence their log values are zero. Each point is the average of 24 one-year simulations.

We next investigated the effects of varying our transmissibility parameter *Q* and the effects of varying the likelihood of scabies importation, quantified by *J* (Fig. 6). While the expected monotonic increase in scabies burden with either increased transmissibility or importation probability was indeed found, there are three important conclusions: first, there exists a non-linear relationship between the magnitude of either variable and the extent of scabies burden. This feature is demonstrated in Fig. 6a and is characterised by the almost linear relationship between scabies prevalence and the natural logarithm of the transmissibility. The non-linearity is striking near baseline where halving *Q* reduces childhood scabies prevalence sixfold. Second, scabies importation only contributes to significant prevalence and incidence rates when the magnitude of *J* is unreasonably high (Fig. 6b). Finally, prevalence and incidence rates in the childhood cohort (necessarily a subset of the whole population) may at times be *negatively correlated* with respect to variation in *Q* or *J*: this characteristic was only found in scenario 2 and is shown in Fig. 6c and 6d. We discuss the relevance of this latter subtle finding in the context of epidemiologic research in the next section.

### Varying the treatment regime

We then varied our treatment protocol, using both density and frequency as key variables (Table 3). As expected, prevalence rates fall when treatment density increases (keeping treatment frequency fixed), or, as treatment frequency increases (keeping treatment density fixed). However, we have constructed Table 3 such that for the main left-to-right diagonal in both scenarios, the product of treatment density and frequency is constant (note that for the diagonal entries in scenario 1 the effective treatment densities are not identical despite equal index case treatments; as noted above this is because all first degree contacts of the index case are also treated). Surprisingly, we found that given the same or similar number of effective treatments, it may be more efficacious to treat scabies in communities as *frequently as possible*. This result is illustrated in scenario 1 by contrasting the outcome of treating 12 index cases and all first-degree contacts every 20 days with the outcome of treating 36 index cases and all first-degree contacts every 60 days (Table 3). Although the total number of effective treatments are equal, the prevalence of scabies for all age groups in the 20-day treated cohort is only 25% of the cohort treated every sixty days. Inspection of the diagonal entries for scenario 1 reveal that scabies prevalence in the cohort treated every five days is only 61% of the corresponding prevalence in the cohort treated every 180 days, despite the same number of effective treatments (Table 3).

### Treating index cases and first degree contacts versus the treatment of index cases only

Finally, we compared our different management regimes – the treatment of the index cases and all first-degree contacts versus the treatment of index cases only – by controlling for all variables. Here we compared the mean prevalence and effective treatment rates for scenario 1 (Table 3; first column, second row box) with scenario 2 (now with the same value of *Q* as scenario 1), utilising the respective results of 24 independent one-year simulations. With similar overall average effective treatment rates (43 and 36 per year for scenarios 1 and 2 respectively) we found similar overall prevalence rates (29% and 32% for scenarios 1 and 2 respectively). These results indicate that when all variables are controlled, including the frequency of treatment administration, the key factor that determines scabies burden is the *effective treatment count.* The method of identifying affected individuals is less important. We discuss the trade-off between the treatment of unaffected individuals and the potential difficulties in the random identification of affected individuals in the next section.

### Box 1

#### Mean-field treatment density and frequency analysis

Can we make predictions regarding scabies prevalence using an analytic model? One method is to assume all individuals are randomly mixing and are equivalent – a mean field approach – and then compare the results with the stochastic simulations. In this Box, we consider a mean field model of the scabies contagion, comparing its predictions with the Monte-Carlo results. Failure of concordance between the two models is likely to be due to stochastic, individual and spatial effects.

**Figure 7:**
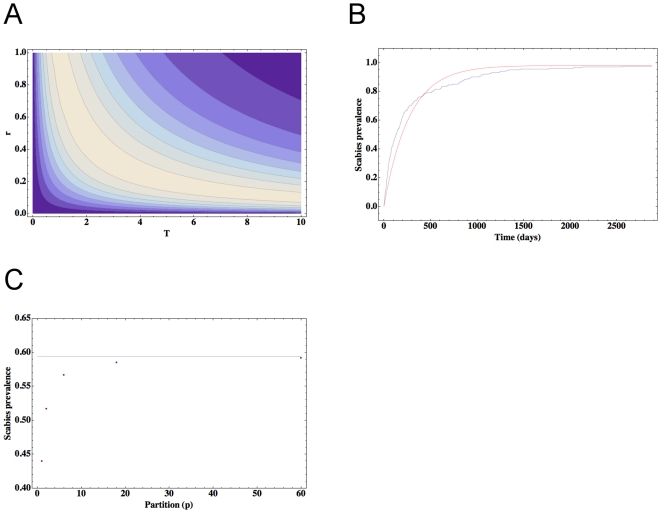
The effect of treatment partitioning on scabies burden. Contour plot (a) (with lighter shades corresponding to larger values) of the maximal possible increase in scabies burden as a function of *r* and *T*. While the global maximum is constant, this maximum is only satisfied for particular choices of *r* and *T*. In (b) we run scenario 2 without treatment from *N* = 0 at *t* = 0 until *N ∼ N_0_* (blue line). The mean field approximation is given by the red curve with rescaled parameter values *N_0_* = 0.98 and *r* = 0.0037 (see equation 1, Box 1). In (c), we plot scabies prevalence for our mean field model as a function of the partitioning *p*, where the *p* are given by 1, 2, 6, 18 and 60. Note scabies prevalence asymptotes to the horizontal line corresponding to the calculated limit as *p* approaches infinity.

We consider the prevalence rates of scabies in a large population subject to two treatment protocols: first, the treatment of *N_T_* individuals at time intervals *t  =  T*, and second, the treatment of *N_T_/p* individuals at time intervals *t  =  T/p*, where *p* is a positive integer. By construction, both regimes treat *N_T_* individuals during time *t  =  T*. For simplicity, we approximate the dynamics of a scabies contagion of magnitude *N* relaxing to steady state *N_0_* with an analytic function of the form:
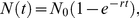
(1)where *N_0_* and *r* are positive constants and 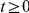. Fig. 7c gives an example of (1) approximating the prevalence time-course of scabies for scenario 2 without treatment (the *relaxation curve*).

We perturb a scabies contagion of magnitude *N* with *N_T_ < N* treatments at *t* = 0. The average number of affected individuals over the time interval *(0, T)* is then given by the integral:
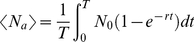
(2)which evaluates to
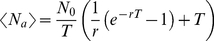
(3)for any choice of *T.*

We now partition the interval *(0, T)* into *p* segments of equal duration. Beginning with the perturbation of a contagion of magnitude *N* at *t* = 0, at each time step *t_j_  =  j T/p* where *j  =  (0, 1, 2, 3… p - 1)* we administer *N_T_/p* treatments. Hence over the time interval *(0, T)* there are *N_T_* treatments administered; this is the same as the non-partitioned case. Note that for *0< t < T* and for any *N_T_ < N*, *r >0* the sign of *N* is always positive.

The average number of affected individuals over the time interval *(0, T)* is then given by the sum of integrals:
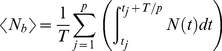
(4)where the *t_j_* define the limits of integration and are given by the solutions of the following equations:
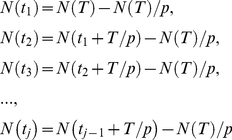
(5)


Symbolic integration and algebraic manipulation of (4) yields solutions of the form
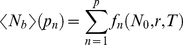
(6)for any choice of partition *p_n_*. The general solution for (4) as a function of *p* is given by the following

expression:
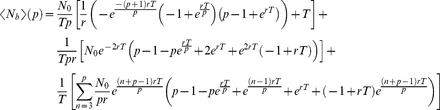
(7)where only the first term is used for the case *p* = 1. Note that in this case equation (7) reduces to equation (3).

We can derive an expression for 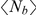 as *p* goes to infinity:
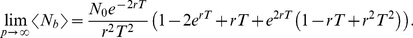
(8)


Finally, we subtract equation (3) from equation (8) yielding an expression for the maximal possible change *S* in scabies burden relative to the non-partitioned case as a function of *r* and *T*:
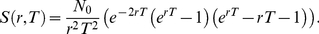
(9)


Equations (7) and (9) reveal two main features: first, since equation (7) is monotonically increasing for any choice of *r* and *T*, scabies burden always increases with larger values of *p*, and second, for any choice of *T*, the global maximum of *S* is fixed at ∼0.16 *N_0_*. Here the value of *r* that is associated with this global maximum will depend on the choice of *T*. Fig. 7a shows a contour plot of (9) for 0*<r<*1 and 0 *<T<*10 where the lighter colours correspond to values of greater magnitude. We conclude that the mathematical model yields prevalence rates that increase as partitioning increases.

#### Example: application to scenario 2

We now compare results derived from this model with results generated from our Monte-Carlo simulations where we fit *N_0_* and *r* in equation (1) to the relaxation curve generated by running scenario 2, without treatment, from *N* = 0.005 at *t* = *0* to *N ∼ N_0_*. We note that while the curve fit is not perfect, it is nonetheless reasonably accurate (Fig. 7b).

We choose *T* = 360 days, *N_T_* = 180 persons and partitions *p* = 2, 6, 18 and 60 hence our treatment frequencies are given by *t* = 180, 60, 20 and 6 days, and the respective treatments densities are given by 90, 30, 10 and 3. Note that, for example, the case *p* = 60 corresponds to the Monte-Carlo simulations for scenario 2 where we treat 3 cases every 6 days, while the case *p* = 2 corresponds to scenario 2 where we treat 90 cases every 180 days (Table 3). Fig. 7c shows mean scabies burden plotted as a function of *p* where the values *N_0_* = 0.98 and *r* = 0.0037 are used in equation (1).

Fig. 7c reveals an increase in scabies prevalence from 52% to 59% from *p* = 2 to *p* = 60. Substitution of the parameter values into the right hand side of equation (8) gives the rescaled limit as *p* approaches infinity; the calculated value is 0.594 and is shown as the dashed horizontal line in Fig. 7c. However, examination of the diagonal entries of scenario 2 in Table 3 reveals that mean scabies prevalence remains at ∼44% as treatment partitioning increases. Hence the increase in prevalence rates with increased partitioning found in the mean-field model is not seen in the stochastic model. We discuss possible causes and implications of this result in the Discussion.

## Discussion

Due to heightened awareness among health workers of the significant cardiac and renal morbidity associated with high scabies burdens among affected communities, the problem has received renewed attention and been approached in two ways: first, by attempting to understand the epidemiologic factors within communities that promote high levels of contagion, and second, by investigating the effects of therapeutic intervention. However, as far as we are aware, ours is the first epidemiologic simulation of the human scabies contagion.

Our results indicate that within affected communities there are a number of key factors that may synergise in promoting high scabies burdens. These include a high average network degree, significant network clustering, and high levels of inter-person transmissibility. While these factors will act synergistically in many communities, it is clear that not all are necessary for scabies burdens to be high. For example, the Kuna Indians observe good personal hygiene; while this is reflected in a low inter-personal transmissibility, overcrowding and significant community network clustering nonetheless predispose to high levels of contagion. Can any of these fundamental community characteristics be addressed? Compared to scabies, there exist many other arguably more significant transmissible diseases among communities affected by poverty and overcrowding: leprosy, cholera and tuberculosis for example – so the prospect for social change to be driven by the scabies problem alone is small. However, we have demonstrated that there are significant non-linear effects on scabies prevalence with respect to *Q* and *J*. For example, the non-linearity suggests that small reductions in the transmissibility *Q*, which could be facilitated by simple education and awareness programs, are likely to yield significant reductions in scabies burdens.

### A stationary process

Our results suggest that it is possible to consider the dynamics of scabies in affected communities as a *stationary process*; this implies its statistical properties are invariant with respect to time (Fig. 4b). This feature contrasts sharply with other transmissible human diseases such as influenza or AIDS [25], [26]. In the former, the process is not stationary because recovered individuals are refractory to reinfection, while in the latter the process is not stationary because HIV acquisition is usually irreversible. The stationary nature of the dynamics of endemic scabies is reflected in its lack of sensitivity to the degree distribution. In contrast to, for example, influenza, where highly connected individuals may play an important role in the spread of disease, hubs are less important in scabies spread because scabies persists until treated and because the probability of acquisition, if continuously exposed to an affected individual, is cumulative over time.

The stationary nature of endemic scabies offers hope that it may be possible to intervene and keep the system at a new steady state – one in which the prevalence rates are much lower. However, in the absence of an effective vaccination, and with scabies continually imported to communities from non-local contacts, it also suggests eradication is impossible and that open-ended treatment regimes are mandatory. This latter point is the key – as noted in the Introduction, many intervention protocols, for various reasons, cannot be maintained indefinitely. When interventions cease, prevalence rates quickly escalate.

### Treatment protocols

Examination of Table 3 reveals that the most effective method of achieving the greatest reductions in scabies burden is to treat all individuals with scabies as frequently as possible. This finding is in accord with views reported elsewhere, where it has been argued that mass screening and treatment of all affected individuals at regular intervals is the most appropriate intervention [4], [14]. Examination of Table 3 reveals an additional benefit: while the prevalence of scabies is kept to a minimum, the number of effective treatments required is not much greater than protocols where the schedule calls for smaller numbers of affected individuals to be treated and where scabies prevalence may be much higher. The explanation of this observation is simple: since the first or second treatment schedule has eliminated most scabies from the community, only a *small fraction* of the large number of scheduled treatments is necessary at each subsequent intervention.

Citing a specific example, it has been argued that other treatment protocols, such as the treatment of smaller numbers of randomly selected affected individuals, is ineffective [14]. However, examination of the diagonal entries of Table 3 for scenarios 1 and 2 reveal that the method of patient selection and the frequency of treatment administration does not matter provided the *same* number of effective treatments, over an identical time period, are administered. In fact, there are three potential advantages in treating smaller numbers of randomly identified affected individuals more frequently.

First, for a given high frequency treatment protocol, low treatment densities are associated with maximal possible reductions in scabies burden. Examination of the first row of Table 3 for scenario 2 reveals that the treatment schedules involving more than 10 affected individuals every six days does not reduce scabies prevalence below 0.02. The maximum benefit is thus realised without the need for further increases in treatment density. In contrast, examination of the last row of Table 3 for scenario 2 reveals that when treatment is administered every 180 days progressive increases in treatment densities are associated with progressively lower scabies burdens.

Second, due to public infrastructure and financial constraints, it may be easier to treat smaller numbers of individuals more frequently than all affected individuals less frequently, since the former avoids the difficulties associated with implementing regular mass screening.

Finally, and as noted in the Results section, when comparing treatment protocols based on frequency, and given the same effective treatment and incidence rates, the prevalence may be *lower* in the cohort treated more frequently. Although this result is not universal (see Table 3), we note that it is counterintuitive and contradicts the results obtained from the mean-field model presented in Box 1. Why is there discord between the two models? The difference in behaviour is likely to be due to the inaccuracy of the mean-field model in reproducing the rates of change of scabies prevalence as a function of scabies prevalence. This inaccuracy is evident in Fig. 7b: although the fit is reasonably good, the mean-field model does not precisely match the relaxation dynamics of the stochastic model. These findings suggest stochastic effects, community structure and the heterogeneity of individuals play an irreducible and non-trivial role in the relaxation dynamics, and are thus likely to be responsible for the different outcomes between the mean-field and stochastic approaches. It is therefore unlikely that a single tunable analytic model can fully capture, with precision, the complexity of a potentially large set of possible stochastic relaxation curves. We conclude that our mean-field model yields an incorrect conclusion – prevalence rates do not increase with increasing partitioning of treatments.

Returning to the Monte-Carlo model, we note that the lower prevalence rates found in cohorts treated more frequently is not necessarily dependent on the treatment of all first-degree contacts of the index case; in addition to scenario 1, the effect also occurs in scenario 2 where only index cases are treated. The caveat associated with treating smaller numbers of affected individuals more frequently is that if scabies burden is initially high, it may take an extended period of time before lower prevalence rates are achieved. For our model, the relaxation times are of the order 360 days; we can therefore expect that it may take a year before steady-state lower prevalence rates are realised. However, this delay is offset by noting that a protocol that treats smaller numbers of individuals more frequently is more likely to be continued for a longer duration in comparison with protocols that specify mass screening and treatment of larger numbers of affected individuals less frequently.

### Treat all first-degree contacts?

Is it necessary to treat all first-degree contacts of index cases? We have shown that the critical factor in determining scabies prevalence is the effective treatment rate, not the method of identifying affected individuals. The advantages of treating all first degree contacts of the index case are clear: there is a higher likelihood of finding affected individuals among household contacts in comparison with random selection, and at least temporarily, scabies may be cleared from a particular household. The disadvantages are also clear: the treatment of individuals without scabies is wasteful, and compliance issues associated with a lack of supervision or poor motivation can undermine its effectiveness [15].

### Modelling communities

From a practical perspective, our results suggest that it may be possible to model any particular community given prior knowledge of the prevalence and either the incidence or treatment rates. It may then be feasible to estimate *Q*, from which a treatment protocol can be designed that will be associated with a pre-defined *target prevalence.* For example, in scenario 2 we see that at baseline, which corresponds to a community of 2000 individuals receiving 30 treatments per week, the prevalence rates are stable at approximately 50%. If we choose a target prevalence of 2%, then examination of Table 3 reveals that we would need to schedule 100 treatments every 6 days if all other community variables were unchanged. However, we find that soon after implementation of this schedule the prevalence drops to low levels hence not all the scheduled effective treatments can be administered. Over the course of 12 months at equilibrium, an average of only 70 effective treatments are required every 6 days. The dynamics of scabies contagion is thus highly non-linear and can therefore be exploited to advantage – in this case an approximate doubling of the effective treatment rate leads to a scabies prevalence *one twenty-fifth* of its previous value. If there are constraints on treatment availability, our results suggest that all first-degree contacts do not need to be treated. Since it is the effective treatment rate that determines scabies burden, lower prevalence rates could be realised with the same treatment supply by targeting affected individuals only.

### Scabies dynamics

We now focus on the dynamics of the scabies contagion. We first note that while distinct communities may have the same prevalence, their respective incidence rates may be different, hence the incidence rate alone is not a good measure of scabies burden. In general, for communities with different values of *Q*, prevalence and incidence rates will both be of greater magnitude with larger values of *Q*. Importation of scabies is likely to be significant only in the context of preventing eradication of the contagion; unless the importation rates are exceptionally and unrealistically high, this factor alone contributes very little to prevalence rates.

As noted in the Results, prevalence and incidence rates in the childhood cohort (necessarily a subset of the whole population) may at times be *negatively correlated* with respect to variation in *Q* or *J*: this characteristic is only found in scenario 2 and is shown in Fig. 6c and 6d. How can this occur? The key factor is the difference between the two scenarios with respect to their treatment regimes: in contrast to the treatment rates in scenario 1, the treatment rates in scenario 2 have a *fixed* upper limit. If the treatment rate is not *a priori* determined exactly (as occurs in scenario 1 where the index case and all first degree contacts are treated) childhood treatment rates increase in proportion to childhood prevalence rates. In contrast, when prevalence rates are high and the upper treatment rate is fixed the treatment rate in the childhood group will be proportional to the *ratio* of childhood to community-wide scabies prevalence. Although for both treatment strategies this ratio is found to decrease as prevalence rates increase, it is only influential in scenario 2 where it leads to a *reduction* in the childhood treatment rates. Since the system is stationary a reduction in the childhood scabies treatment rate is causally linked with a reduction in the incidence rate. We conclude that if the effective treatment rate is fixed, and if treatments are given to randomly affected individuals of any age, the incidence rate of scabies in a childhood cohort may be lower in a community with a higher value of *Q* when compared to the incidence rate of childhood scabies in a community with a lower value of *Q*. From an epidemiologic perspective, this counterintuitive result suggests that measuring the incidence rate in childhood cohorts may not be a good measure of scabies transmissibility within the community as a whole.

### Recommendations

While scabies remains a significant public health problem worldwide, we believe all is not lost. We can identify three unsatisfactory situations where therapeutic intervention protocols, guided by our findings, may lead to reduced and potentially acceptable long-term scabies burdens.

First, while it is clear that mass screening and treatment of all affected individuals will lead to the greatest reductions in scabies prevalence, it is very difficult for such protocols to be maintained over many years. If the protocol is terminated, the outcome can be considered a failure since prevalence rates quickly escalate. Many factors may contribute to the cessation of such protocols: unforeseen external events, financial constraints, community apathy as scabies burdens drops, and community resentment at the intermittent but all too regular social disruption deemed necessary by ‘foreign’ health care workers. The abrupt termination of the successful intervention implemented among the Kuna Indians in 1989 is an example of such a situation [14]. Second, we consider the situation where a smaller number of treatments are administered more frequently. As noted above, one particularly unsatisfactory outcome reported was the situation where, among a population of 2000 individuals, the treatment of 30 cases per week for two years did not reduce scabies prevalence below 50% [14]. In this example there is clearly great effort but minimal benefit. Finally, there exist communities with hyper-endemic scabies where no active intervention exists. Here a lack of funding and general pessimism among health care workers may preclude any attempt to develop either a scabies control policy or an intervention strategy.

Guided by the insights gained from our simulations, we suggest a potentially fruitful approach to these unsatisfactory situations is to treat a pre-determined number of randomly chosen affected individuals more frequently. For the first example, field data provide the necessary information that permit the design of a treatment protocol that will satisfy any pre-defined target prevalence. In this case low-density treatment protocols are less likely to be adversely affected by unforeseen external events, are less patronising and should produce less community disruption than community-wide ‘scabies days’. For the second example, the despair associated with this situation is largely unfounded: we have shown in the previous section that doubling the effective treatment rate reduces long-term scabies prevalence from 50% to 2%. Doubling the effective treatment rate in this case is extremely beneficial and does not even require twice the effort since the infrastructure to deal with a significant patient load must already exist. Granted, the third example requires an epidemiologic survey before any modelling can be undertaken. However, in some cases – due to the existence of non-linearity – the results of applying our model may lead to the realisation that, for a given treatment frequency, treatment densities do not have to be impractically high to produce significant reductions in scabies burden. It may then be possible to gain confidence in the outcome of implementing such a protocol.

We acknowledge that there may be difficulties in implementing frequent treatment strategies that are required to meet treatment density objectives. Since in our simulations we selected index cases for treatment at random, problems may arise with reproducing a random selection process within a real community. In the identification of affected individuals, while schools and health clinics should provide enough cases to approximate a random selection process, it is clear that recalcitrant individuals may escape detection. However, since the protocol is designed to operate for many years, it is expected that all affected individuals will eventually be found and treated. If the infrastructure exists, all first-degree contacts of affected individuals should be screened, and if positive for scabies, treated. While the parents of children who associate with affected children at school should also be screened, this does not have to undertaken with any urgency. Rather, these parents are likely to provide a high-yield source of affected individuals that can help meet low-density treatment objectives over an extended period. The method will require community-wide participation and a health care worker who can monitor treatment rates. If difficulties arise in finding enough affected individuals to meet treatment objectives, then we can be confident that the protocol is working since this difficulty is likely to be due to low prevalence rates.
